# Sildenafil Recovers Burn-Induced Cardiomyopathy

**DOI:** 10.3390/cells9061393

**Published:** 2020-06-03

**Authors:** Jake J. Wen, Claire Cummins, Ravi S. Radhakrishnan

**Affiliations:** Department of Surgery, University of Texas Medical Branch, 301 University Blvd., Galveston, TX 77555, USA; cbcummin@utmb.edu

**Keywords:** burn injury, sildenafil, cardiomyopathy, PDE5A, oxidative stress, fibrogenesis

## Abstract

**Background:** Severe burn injury initiates a feedback cycle of inflammation, fibrosis, oxidative stress and cardiac mitochondrial damage via the PDE5A-cGMP-PKG pathway. **Aim:** To test if the PDE5A-cGMP-PKG pathway may contribute to burn-induced heart dysfunction. **Methods:** Sprague–Dawley rats were divided four groups: sham; sham/sildenafil; 24 h post burn (60% total body surface area scald burn, harvested at 24 h post burn); and 24 h post burn/sildenafil. We monitored heart function and oxidative adducts, as well as cardiac inflammatory, cardiac fibrosis and cardiac remodeling responses in vivo. **Results:** Sildenafil inhibited the burn-induced PDE5A mRNA level and increased the cGMP level and PKG activity, leading to the normalization of PKG down-regulated genes (IRAG, PLB, RGS2, RhoA and MYTP), a decreased ROS level (H2O2), decreased oxidatively modified adducts (malonyldialdehyde [MDA], carbonyls), attenuated fibrogenesis as well as fibrosis gene expression (ANP, BNP, COL1A2, COL3A2, αSMA and αsk-Actin), and reduced inflammation and related gene expression (RELA, IL-18 and TGF-β) after the burn. Additionally, sildenafil treatment preserved left ventricular heart function (CO, EF, SV, LVvol at systolic, LVPW at diastolic and FS) and recovered the oxidant/antioxidant balance (total antioxidant, total SOD activity and Cu,ZnSOD activity). **Conclusions:** The PDE5A-cGMP-PKG pathway mediates burn-induced heart dysfunction. Sildenafil treatment recovers burn-induced cardiac dysfunction.

## 1. Introduction

Globally, burns are a serious public health problem. An estimated 265,000 deaths occur each year from fires alone, with more deaths from scalds, electrical burns and other forms of burns, for which global data are not available [1]. Every day, over 300 children aged 0 to 19 are treated in emergency rooms for burn-related injuries, and two children die as a result of being burned [2]. In the United States, burns are also a considerable health problem, with 500,000 injured people, resulting in more than 40,000 hospitalizations, 4000 deaths and more than $18 billion in costs each year [3,4]. Burn injury causes hemodynamic derangements and compromised heart function, leading to organ hypoperfusion, burn zone extension and increased susceptibility to wound infection [5].

In cardiomyocytes, atrial natriuretic peptide (ANP) and nitric oxide (NO) produce cyclic guanosine monophosphate (cGMP) by activating guanylyl cyclase (GC), which stimulates cGMP-dependent protein kinase (PKG1α) [6]. PKG1α maintains the contractile force of cardiomyocytes and phosphorylates serine and threonine residues on numerous cytosolic proteins [7]. The cGMP-PKG axis regulates the activation of phosphodiesterase, which hydrolyzes cyclic nucleotide monophosphate (cNMP) [8]. While phosphodiesterases (PDEs) have seven isoforms in myocytes (including PDE1, 2, 3, 4, 5, 8, and 9), PDE5A is cardiomyocyte-specific, and functions to hydrolyze cGMP and negatively regulate cardiomyocyte inotropy [9,10]. Recent studies indicate that PDE5A subcellularly localizes to cardiomyocyte z-bands [11], suggesting that PDE5A might modulate function in the stressed heart. In addition, PDE5A inhibitors, such as sildenafil, prolong the effect of NO and cGMP, causing increased vasodilation through decreased smooth muscle tone [12]. In our preliminary work, we observed that burn-induced cardiac mitochondrial dysfunction is modulated via the AMPK-SIRT1-PGC1α-NFE2L2 ARE pathway [13] and the cGMP-PKG pathway [14]. However, little is known regarding the specific role of the PDE5A-cGMP-PKG1α pathway in burn-induced cardiac dysfunction.

In this study, we explored the role of the PDE5A-cGMP-PKG pathway in the pathogenesis of burn-induced cardiac dysfunction by studying inflammation, fibrogenesis, oxidative stress and heart function. In addition, we studied if sildenafil administration would attenuate burn-induced cardiac dysfunction.

## 2. Materials and Methods

### 2.1. Ethics Statement

All animal research procedures abide by the National Institutes of Health guidelines for experimental animal use, and were approved by the Institutional Animal Care and Use Committee at the University of Texas Medical Branch (Galveston, TX. Protocol number: 1509059, 31 August 2015).

### 2.2. Animals and Burn Model

Male Sprague–Dawley rats were purchased from Harlan Laboratories (Indianapolis, IN). The animals were allowed to acclimate for one week before experimentation, and received food and water ad libitum throughout the study. The animals were kept on a 12:12 light–dark cycle at ~25 °C. A well-established model for the induction of a 60% total body surface area (TBSA) full-thickness burn was used [15,16]. In detail, the rats (300–350 g) were administered buprenorphine (0.05 mg/kg s.c.) for analgesia, and anesthetized with isoflurane (3–5%). To induce a scald burn, the rats were placed within a protective frame mold that exposed ~30% TBSA and submerged on both the dorsal and ventral sides in 95–100 °C water, resulting in 60% TBSA burn in total, as we have previously published [13]. Lactated Ringer (LR) solution (40 mL/kg, i.p. ± sildenafil, 2 mg/kg body weight) was injected immediately after the burn for resuscitation, and the rats received oxygen during the recovery from anesthesia. Analgesia (buprenorphine, 0.05 mg/kg) was given as needed every 6 h after the burn. Due to damage to nerve endings in full-thickness burns, additional analgesia was rarely required. For sham control, animals underwent all the same procedures as those in the burn group, except for submersion in room temperature water instead of 95–100 °C water. At pre-determined time points, rats were humanely euthanized by bilateral thoracotomy under anesthesia (≥5% isoflurane), and left ventricle tissue was collected to be stored at −80 °C.

### 2.3. Nano LC-MS/MS

Sample digestion: Approximately 45 µL of heart tissue lysates (50 µg protein), made in the tissue lysate buffer (5% SDS, 50 mM Triethylammonium bicarbonate (TEAB); pH 7.55), were reduced in 10 mM DTT at 65 °C for 10 min, then cooled to room temperature (RT). We then added 3.75 µL of 1 M iodoacetamide for 20 min in the dark, after which 0.5 µL of 2M DTT was added to quench the reaction. 5 µL of 12% phosphoric acid and 350 µL of binding buffer (90% Methanol, 100 mM TEAB final; pH 7.1) were added to the solution and passed through an S-Trap spin column (ProtiFi.com) for 30 s at 4000 g, and then washed with 400 µL of binding buffer for three times. Trypsin was added (1:25 ratio in 50 mM TEAB, pH = 8) and incubated at 37 °C for 4 h. Peptides were eluted with 80 µL of 50 mM TEAB, followed by 80 µL of 0.2% formic acid, and finally 80 µL of 50% acetonitrile, 0.2% formic acid. The combined peptide solution was then dried in a speed vac and resuspended in 2% acetonitrile, 0.1% formic acid. NanoLC MS/MS Analysis: Peptide mixtures were analyzed by nanoflow liquid chromatography tandem mass spectrometry (nanoLC-MS/MS) using a nano-LC chromatography system (UltiMate 3000 RSLCnano, Dionex, Sunnyvale, CA, USA) coupled online to a Thermo Orbitrap Fusion mass spectrometer (Thermo Fisher Scientific, San Jose, CA, USA) through a nano-spray ion source (Thermo Scientific). The analytical column was an Acclaim PepMap 100 (75 µm × 25 cm) from Thermo Scientific. After equilibrating the column, the samples (1 µL in solvent A) were injected onto the trap column and subsequently eluted (400 nL/min) by gradient elution onto the C18 column. All LC-MS/MS data were acquired using XCalibur, version 2.1.0 (Thermo Fisher Scientific), in positive ion mode, using a top speed data-dependent acquisition (DDA) method with a 3 s cycle time. The survey scans (*m*/*z* 350–1500) were acquired in the Orbitrap at 120,000 resolution (at *m*/*z* = 400) in profile mode, with a maximum injection time of 50 ms and an AGC target of 400,000 ions. The S-lens RF level was set to 60. Isolation was performed in the quadrupole with a 1.6 Da isolation window, and CID MS/MS acquisition was performed in profile mode using a rapid scan rate with detection in the orbitrap (res: 35,000) with the following settings: parent threshold = 5000; collision energy = 35%; maximum injection time 100 ms; AGC target 500,000 ions. Monoisotopic precursor selection (MIPS) and charge state filtering were on, with charge states 2–6 included. Dynamic exclusion was used to remove selected precursor ions, with a ±10 ppm mass tolerance, for 60 s after acquisition of one MS/MS spectrum. Database Searching: Tandem mass spectra were extracted and charge state deconvoluted with Proteome Discoverer (Thermo Fisher, version 1.4.1.14). All MS/MS spectra were searched against a Uniprot Rattus database (version 05-16-2017) using Sequest. Searches were performed with a parent ion tolerance of 5 ppm and a fragment ion tolerance of 0.60 Da. Trypsin was specified as the enzyme, allowing for two missed cleavages. Fixed modification of carbamidomethyl (C) and variable modifications of oxidation (M) and glycosylation were specified in Sequest.

### 2.4. cGMP Level

Weighted heart tissues were homogenized on ice (5–10 mL of 5% trichloroacetic acid (TCA)/per gram of tissue). After centrifugation at 1500× *g* for 10 min, TCA was extracted five times from the supernatant with water saturated ether. The aqueous phase was dried under a stream of nitrogen and resuspended in 1.5 mL of phosphate buffer. cGMP levels were measured by ELISA (variability among triplicate values, 10%). The values of cGMP in blank were subtracted, and the results were expressed as pmol/mg for tissues.

### 2.5. The cGMP Dependent Protein Kinase (PKG) Activity

A CycLex^®^ cGK (PKG) ELISA Assay Kit (MBL International Corp, Woburn, MA, USA) was employed to measure the PKG activity. Briefly, tissue homogenates (10 µg protein/10 µL) were added to 96-well plates pre-coated with histone H1 peptide containing threonine residues, and sequentially incubated for 30 min in the presence of cGMP and ATP, and then for 60 min with HRP-conjugated anti-phospho-G-kinase substrate threonine 68/119 monoclonal antibody. The plates were then washed, and HRP catalyzed conversion of chromogenic TMB substrate to blue color was recorded at 450/540 nm (standard curve: 1–10-units recombinant cGK [PKG] protein).

### 2.6. Echocardiography (ECHO)

Rats were sedated with inhalant anesthesia (1.5% isoflurane/100% O_2_) and placed supine on an electrical heating pad at 37 °C, and heart rate and respiratory physiology were continuously monitored by ECHO. After shaving the chest, warmed ultrasound gel was applied, and transthoracic ECHO was performed using the Vevo^®^ 2100 ultrasound system (VisualSonics, Toronto, On, Canada) equipped with a high-frequency linear array transducer (MS250 13-24 MHz) [17]. All measurements were obtained in triplicate, and data were analyzed using the Vevo^®^ 2100 standard measurement package.

### 2.7. Histology

Tissue sections were fixed in 10% buffered formalin for at least 24 h, dehydrated in absolute ethanol, cleared in xylene and embedded in paraffin. For whole heart tissue, after extracting blood from the heart, the heart was perfused through the aorta by giving 50 mL of 10% buffered formalin. Five-micron tissue sections were subjected to staining with hematoxylin and eosin (H&E) or Mason’s Trichrome at the Research Histopathology Core at the UTMB. The images were obtained by Motic EasyScanner and analyzed using Motic DSAssistant software (Motic North America, Richmond, British, BC, Canada) and Image J software. Myocarditis (presence of inflammatory cells) in H&E stained tissue sections was scored as 0 (absent), 1 (focal/mild, ≤1 foci), 2 (moderate, ≥2 inflammatory foci), 3 (extensive coalescing of inflammatory foci or disseminated inflammation), or 4 (diffuse inflammation, tissue necrosis, interstitial edema and loss of integrity). Inflammatory infiltrates were characterized as ‘diffuse’ or ‘focal’, depending upon how closely the inflammatory cells were associated [18]. Fibrosis was assessed by measuring the collagen area as a percentage of the total myocardial area, and categorized as (0) <1%, (1) 1–5%, (2) 5–10%, (3) 10–15%, and >15%, based on percentage of fibrotic area [19].

### 2.8. Gene Expression Analysis

Heart tissue-sections (10 mg) were homogenized in 100 µL TRIzol reagent (Invitrogen, Carlsbad, CA, USA), and total RNA was extracted and precipitated by the chloroform/isoamyl alcohol/isopropanol method. The RNAs were treated by DNase I, RNase-free (Westlake, LA, USA. Cat# M0303S) to digest contaminated genomic DNA. To judge the integrity and overall quality of isolated RNAs, 2 µg RNAs were added to 10× native agarose gel loading buffer (15% ficoll, 0.25% xylene cyanol, 0.25% bromophenol blue) and run on 1% native agarose gels. The integrity RNAs were washed with 75% cold ethanol, re-suspended in 20 µL of UltraPure™ nucleotide-free distilled water, and a DU^®^ 700 UV/Visible Spectrophotometer (Beckman Coulter, Pasadena, CA, USA) was used to measure absorbance at 260 and 280 nm (OD_260/280_ ratio ≥ 2, 1 OD_260_ Unit = 40 µg/mL RNA). Total RNA (2 µg) was reverse transcribed with Oligo(dT)_20_ primer using a SuperScript^®^ III Reverse Transcriptase (Invitrogen). The cDNA was used as a template in a real-time quantitative PCR on an iCycler Thermal Cycler with SYBR-Green Supermix (Bio-Rad, Hercules, CA, USA) and gene-specific oligonucleotides (Table 1). The threshold cycle (Ct) values for target mRNAs were normalized to GAPDH/β-actin/α-tubulin mRNAs, and the relative expression level of each target gene was calculated as fold change [19].

### 2.9. Oxidative Stress

The protein carbonyls in the tissue homogenates were measured by colorimetric protein carbonyl assay (Cayman Chemicals, cat# 10005020), according to the instructions provided by the manufacturer. Protein carbonyl content is expressed in nmol/mg of protein. ROS release was measured by Amplex red assay. Briefly, homogenized samples of heart tissue (50 µg protein) were added to black flat-bottom plates. The reaction was initiated by the addition of 50 µL each of 100 μM Amplex red (Invitrogen) and 0.3 U/mL HRP. The HRP catalyzed Amplex red oxidation by H_2_O_2_, resulting in fluorescent resorufin formation, was monitored at Ex_563 nm_/Em_587 nm_ (standard curve: 50 nM–5 M H_2_O_2_) using a Spectra Max M2 microplate reader (Molecular Devices, San Jose, CA, USA). A thiobarbituric acid-reactive substances (TBARS) assay was used to measure MDA-TBA adduct formation using the Cayman TBARS assay kit (cat# 700870). The MDA-TBA adduct was measured colorimetrically at 530–540 nm using a microplate reader (BioTek, Winooski, VT, USA). The MDA concentration is expressed in nM/mg protein.

### 2.10. Antioxidant Levels

Total antioxidant capacity (TAC) was assessed using lag time by antioxidants against the myoglobin-induced oxidation of 2,2′-azino-di-(3-ethylbenzthiazoline)-6-sulfonic acid (ABTS) with H_2_O_2_ (Antioxidant Assay kit, Cayman Chemical, Ann Arbor, MI, USA, cat# 709001). Briefly, 20 µL of plasma samples (diluted 1:20, *v*/*v*) and/or heart homogenates (15 µg protein) were added in triplicate to 96-well plates and mixed with 90 µL of 10 mM PBS (pH 7.2), 50 µL of myoglobin solution and 20 µL of 3 mM ABTS. The reaction was initiated with H_2_O_2_ (20 µL) and changes in color were monitored at 600 nm (standard curve: 2–25 µM trolox). Total SOD activity was measured using a commercially available kit (Superoxide Dismutase Assay Kit, Cayman Chemical, cat# 706002) according to the manufacturer’s instructions. Total SOD activity is expressed as U/mg of protein. Cu,ZnSOD activity in the tissue homogenates was measured by the addition of SDS (final concentration: 2%) into the sample solutions to inhibit MnSOD (standard curve: 0.005–0.05 U/mL recombinant CuZnSOD).

### 2.11. ATP Colorimetric Assay

Tissues were homogenized on dry ice using the ice-cold ATP assay buffer provided as part of the ATP colorimetric Assay kit (Abcam, Cambridge, UK, cat# ab83355;). A 5 μL aliquot of the homogenized tissue was used to determine protein concentrations using the Thermo Scientific BCA method. ATP concentrations in the samples were calculated by plotting the measured optical densitometry at 570 nm in a microplate reader versus the linear distribution generated by the standard curve, with a final adjustment for protein concentration.

### 2.12. Statistical Analysis

All experiments were conducted with triplicate observations per sample (n = 6–9 rats/group) and data were expressed as mean ± standard error mean (SEM). All data were analyzed using GraphPad Prism8.30 software. The data (linear range or log10 transformed) were analyzed by the Kolmogorov-Smirnov test under column statistics to determine if the data are normally distributed. Normally distributed data were analyzed by Student’s *t*-test (comparison of two groups) and one-way ANOVA with Tukey’s test (comparison of multiple groups). If data were not normally distributed, then Mann-Whitney (comparison of two groups) and Kruskal-Wallis (K-W, comparison of multiple groups) tests were employed. Significance is presented by * 24 hpb vs. sham normal or ^&^ 24 hpb vs. 24 hpb/sildenafil (*^,&^ *p* < 0.05, **^,&&^ *p* < 0.01, ***^,&&&^ *p* < 0.001).

## 3. Results

### 3.1. The Importance of the PDE5A-cGMP-PKG Pathway in Cardiomyocytes after a Burn

**Figure 1:**
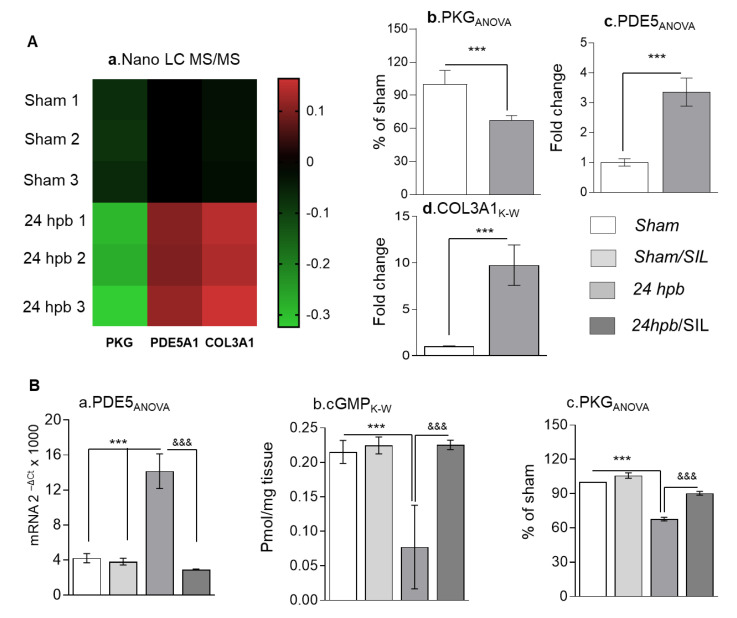
The importance of the PDE5A-cGMP-PKG pathway in cardiomyocytes after a burn. Sprague–Dawley rats were randomized to a 60% TBSA scald burn or sham procedure with standard resuscitation with/without sildenafil (SIL). Heart tissues and blood were harvested at 24 h post burn (24 hpb ± SIL). (**A**) Representative LC MS/MS heat map; green color indicates down-regulated proteins, black color indicates unchanged proteins and red indicates up-regulated proteins (A,a). Corresponding expression levels of PKG (A,b), PDE5A (A,c.) and COL3A1 (A,d). (**B**) Myocardial levels of PDE5 mRNA by qRT-PCR (panel a), cyclic GMP (cGMP) protein levels in heart tissue (panel b) and PKG activity in heart tissue (panel d). Data are presented as mean value ± SD, shown as * (24 hpb vs. matched sham control) or ^&^ (24 hpb vs. 24 hpb/SIL), and presented as ***^, &&&^ *p* < 0.001 (n = ≥ 6 per group).

To know what pathways would be involved in heart dysfunction after a burn, we first employed the Nano LC MS/MS approach to run proteomics (Figure 1(Aa)). Compared to the sham group, the burn group demonstrated a 32% decrease in PKG protein level (Figure 1(Ab)), a three-fold increase in PDE5A protein level (Figure 1(Ac)) and a ten-fold increase in COL3A protein level (Figure 1(Ad)). qPCR found a two-fold increase in PDE5A mRNA level (Figure 1(Ba)). Additionally, we found a two-fold decrease in cardiac cGMP levels (Figure 1(Bb)). Functionally, our data demonstrated a 32% decrease in PKG activity (Figure 1(Bc)). For confirmation of PDE5A-cGMP-PKG pathway involvement, sildenafil was administered after the burn. Sildenafil treatment normalized the levels of myocardial expression of PDE5A, cGMP level and PKG activity (Figure 1(Ba–c)). Altogether, these data suggest that burn-induced heart dysfunction occurs via the PDE5A-cGMP-PKG pathway.

### 3.2. Effect of PDE5A Inhibition on Burn-Induced Cardiac Dysfunction

**Figure 2:**
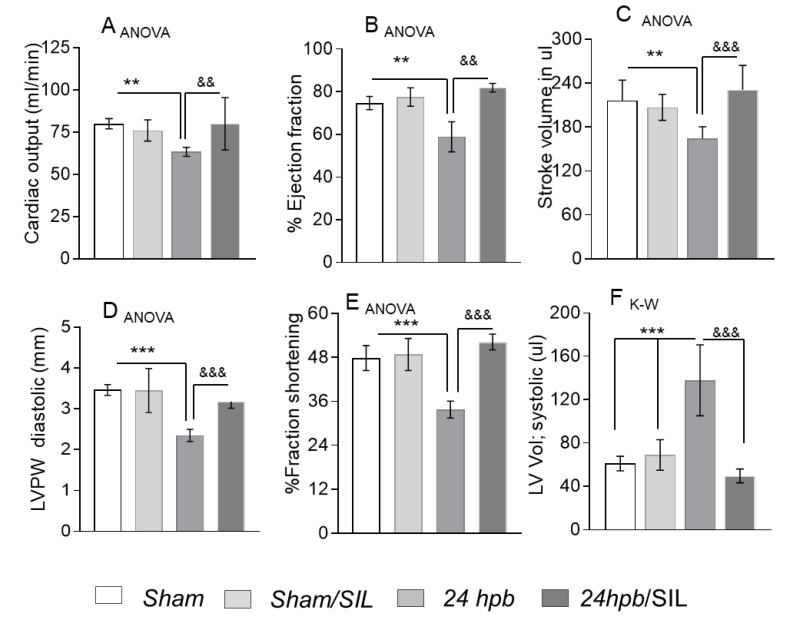
Effect of PDE5A inhibition on burn-induced cardiac dysfunction. Sprague–Dawley rats were randomized to a 60% TBSA scald burn or sham procedure with standard resuscitation with/without sildenafil (SIL). Heart function was measured using ECHO (Vevo^®^ 2100 System) at 24 h post burn (24 hpb ± SIL). Shown are (**A**) cardiac output (CO), (**B**) ejection fraction (EF), (**C**) stroke volume (SV), (**D**) left ventricular posterior wall at diastole (LVPW), (**E**), fractional shortening (FS) and (F) left ventricular systolic volume (LV Vol.). Data are presented as mean value ± SD, shown as ^**, &&^ *p* < 0.01, ***^, &&&^ *p* < 0.001 (n = ≥ 6 per group).

ECHO demonstrated that systolic functions, including cardiac output (CO, Figure 2A), ejection fraction (EF, Figure 2B), stroke volume (SV, Figure 2C), left ventricular posterior wall thickness in diastole (LVPW, Figure 2D) and fractional shortening (FS, Figure 2E), were significantly decreased by 21%, 21%, 24%, 32% and 39%, respectively, after the burn. Left ventricular systolic volume increased by 126% (LV_vol, s_, Figure 2D) after the burn. Treatment with sildenafil preserved all functional cardiac parameters at sham levels (Figure 2). Altogether, these data suggest that burn-induced heart dysfunction is ameliorated by PDE5A inhibition.

### 3.3. Effect of PDE5 Inhibition on Burn-Induced Cardiac Fibrogenesis

**Figure 3:**
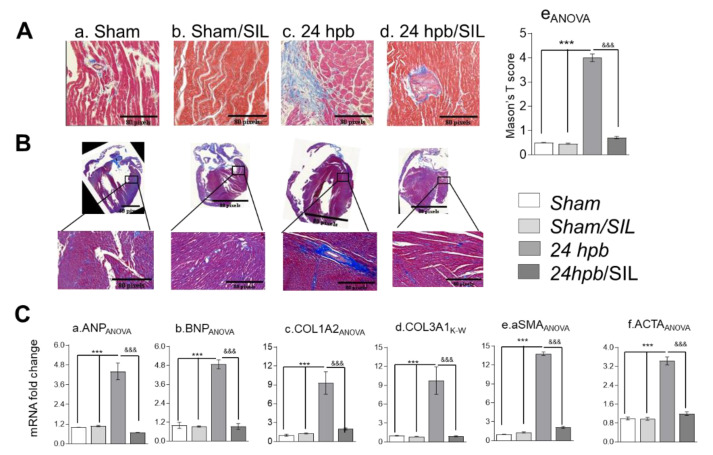
Effect of PDE5 inhibition on burn-induced cardiac fibrogenesis. Sprague–Dawley rats were randomized to a 60% TBSA scald burn or sham procedure with standard resuscitation with/without sildenafil (SIL). Heart tissues and blood were harvested at 24 h post burn (24 hpb ± SIL). (**A**) Representative myocardial fibrosis levels via Masson’s trichrome staining (panels a–d). (**B**) Representative whole hearts stained with Masson’s trichrome and scored for fibrosis (panel e). (**C**) Quantitative RT-PCR analysis of mRNA levels for fibrosis markers. Shown are atrial natriuretic peptide (panel a, ANP), natriuretic peptide B (panel b, BNP), collagen isoforms COLIA2 and COL IIIA1 (panels c & d), actin, alpha, cardiac muscle 1 (aSMA, panel e), and actin, alpha 2, smooth muscle, aorta (panel f). Results were normalized to rat GAPDH and β-actin mRNAs, and represent fold change after the burn (±SIL), as compared to that noted in matched normal controls. In all figures, data are plotted as mean value ± SEM (n ≥ 6 per group). Significance is shown as * (24 hpb vs. matched control) or ^&^ (24 hpb/untreated vs. 24 hpb/SIL), and presented as ***^,&&&^ *p* < 0.001 (n = ≥ 6 per group).

Histologic examination of LV heart muscle pieces indicated a seven-fold increase in collagen accumulation (Figure 3(Aa,c); score: 4.023 ± 0.395 vs. 0.5 ± 0.04). To confirm that these results were not location specific, we fixed a whole heart, cut vertically to show all four chambers, and then performed Trichrome staining (Figure 3B). Whole heart staining for fibrosis demonstrated that the burn injury resulted in cardiac fibrogenesis, as shown in Figure 3. Additionally, qPCR demonstrated approximately four-fold increase in ANP and BNP expression (Figure 3(Ca,b)), an approximately nine-fold increase in collagens I and III expression (Figure 3(Cc,d)), and a greater than three-fold increase in alpha cardiac smooth muscle actin1 (αSMA) and alpha cardiac smooth muscle actin 2 (ACTA) (Figure 3(Ce,f)) after the burn. Sildenafil treatment alleviated more than 90% of collagen deposits after the burn (Figure 3A,B). Sildenafil treatment also normalized the mRNA expression levels of ANP and BNP, collagens I and III, αSMA and ACTA (Figure 3C). These data suggest that treatment with sildenafil maintains the PDE5A-cGMP-PKG balance and has a cardio-protective role via inhibition of burn-induced interruption of the PDE5A-cGMP-PKG pathway.

### 3.4. Effect of PDE5A Inhibition on Myocardial Inflammation after a Burn

**Figure 4:**
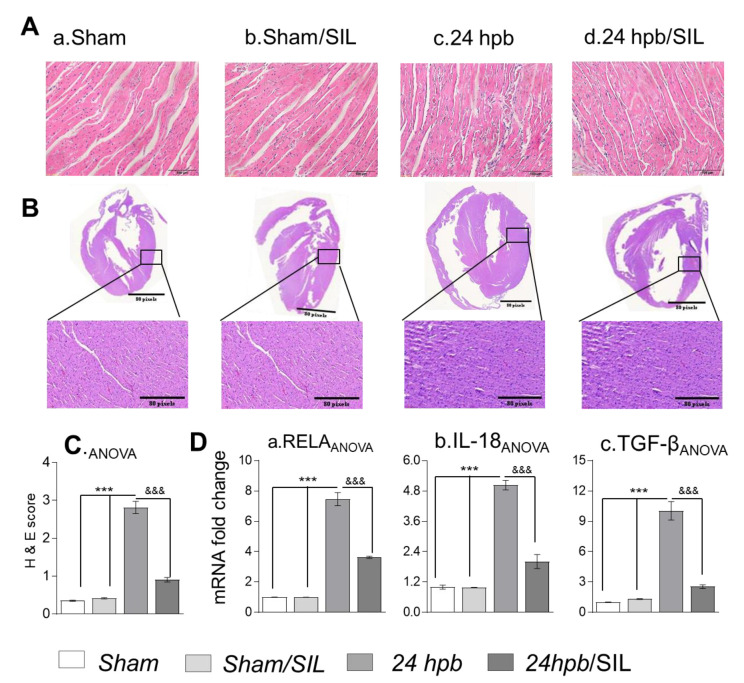
Effect of PDE5 inhibition on myocardial inflammation after a burn. Sprague–Dawley rats were randomized to a 60% TBSA scald burn or sham procedure with standard resuscitation with/without sildenafil (SIL). Heart tissues and blood were harvested at 24 h post burn (24 hpb ± SIL). (**A**) H&E staining of the left ventricle (magnification: 20×. Pink: muscle/cytoplasm/keratin, blue: nuclear) (panels a–d), (**B**) H&E staining of a whole heart (magnification: 20×. Purple: muscle/cytoplasm/keratin, blue: nuclear), and scored for inflammation (**C**). (**D**) Shown are the myocardial levels of inflammation cytokine mRNA levels determined by real time PCR, including RELA (a), IL-18 (b) and TGF-β (c). Results were normalized to rat GAPDH and β-actin mRNAs, and represent fold change after a burn (± SIL), as compared to that noted in matched normal controls. In all figures, data are plotted as mean value ± SD. Significance is shown as * (24 hpb vs. control) or ^&^ (24 hpb vs. 24 hpb/SIL), and data are presented as ***^, &&&^ *p* < 0.001 (n = ≥ 6 per group).

The cardiac inflammatory response plays an important role in the pathogenesis of cardiac tissue damage [20]. PDE5A inhibitors may increase cGMP levels, thus abrogating cardiac inflammation and cellular damage. Histologic studies exhibited diffuse inflammatory infiltrates in the myocardial tissue after the burn (H&E score: 2.812 ± 0.393 vs. 0.35 ± 0.039; Figure 4(Aa, c and C)). This was associated with a greater than five-fold increase in NF-κB and cytokines (RELA, 7.46-fold increase; IL-18, 5.03-fold increase and TGF-β, 10.04-fold increase; Figure 4(Da–c)). Similar to Trichrome staining, H&E staining of a whole heart provided the same results (Figure 4(Ba,c)). Sildenafil significantly reduced myocardial tissue inflammatory infiltrates (histological score: 0.905 ± 0.148; Figure 4(Ad, Bd and C)). NF-κB and cytokine expression was also normalized after sildenafil treatment (Figure 4D). These data suggest that PDE5A inhibition was beneficial in regulating cardiac inflammatory infiltrates and cardiac tissue damage caused by severe burns.

### 3.5. The Effect of PDE5A Inhibition on Downstream Gene Expression after a Burn

**Figure 5:**
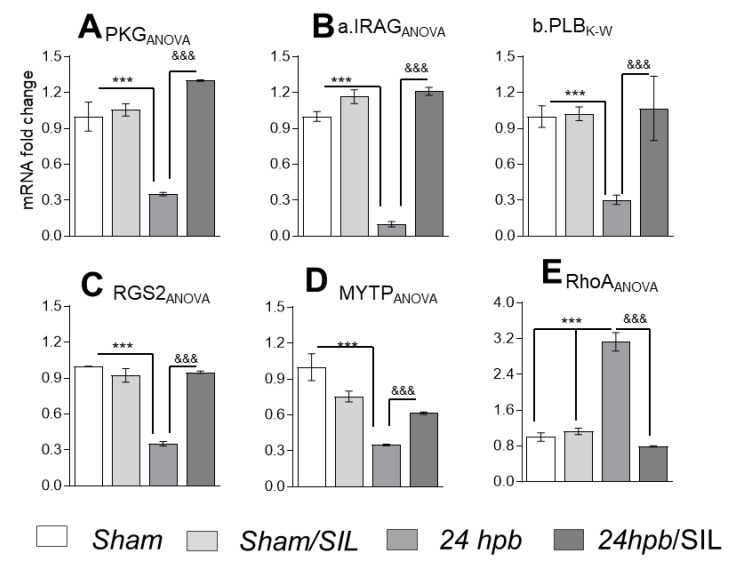
The effect of PDE5A inhibition on downstream gene expression after a burn. Sprague–Dawley rats were randomized to a 60% TBSA scald burn or sham procedure with standard resuscitation with/without sildenafil (SIL). Heart tissues and blood were harvested at 24 h post burn (24 hpb ± SIL). Isolated heart tissue RNAs (RNeasy Mini Kit) were used to synthesize cDNAs (The SuperScript III First-Strand Synthesis System), and mRNA level was measured by qRT-PCR. (**A**) Myocardial levels of PKG mRNA. (**B**) Myocardial levels of cGMP-PKG pathway markers, including IRAG (a) and PLB (b) mRNA levels. (**C**) Myocardial level of RGS2 mRNA. (**D**) Myocardial level of RhoA mRNA. (**E**) Myocardial levels of MYTP mRNA levels. Results were normalized to rat GAPDH and β-actin mRNAs, and represent fold change after a burn (±SIL), as compared to that noted in matched normal controls. In all figures, data are plotted as mean value ± SD. Significance is shown as * (24 hpb vs. control) or ^&^ (24 hpb vs. 24 hpb/SIL), and presented as ***^, &&&^ *p* < 0.001 (n = ≥ 6 per group).

To examine the role of PDE5A-regulated gene expression, we measured PDE5A-regulated gene mRNA levels. We found burn induced decreases of PKG (65%, Figure 5A), IRAG (90% Figure 5(B,a)), PLB (70%, Figure 5(B,b)), RGS2 (65%, Figure 5C) and MYTP (65%, Figure 5D). A burn also induces a two-fold increase of RhoA mRNA levels (Figure 5E). These mRNA levels were normalized after sildenafil administration (Figure 5A–E). These data suggest that PDE5A inhibition was useful in restoring cardiac PKG dysregulation caused by severe burns.

### 3.6. The Effect of PDE5A Inhibition on Oxidant/Antioxidant Imbalance

**Figure 6:**
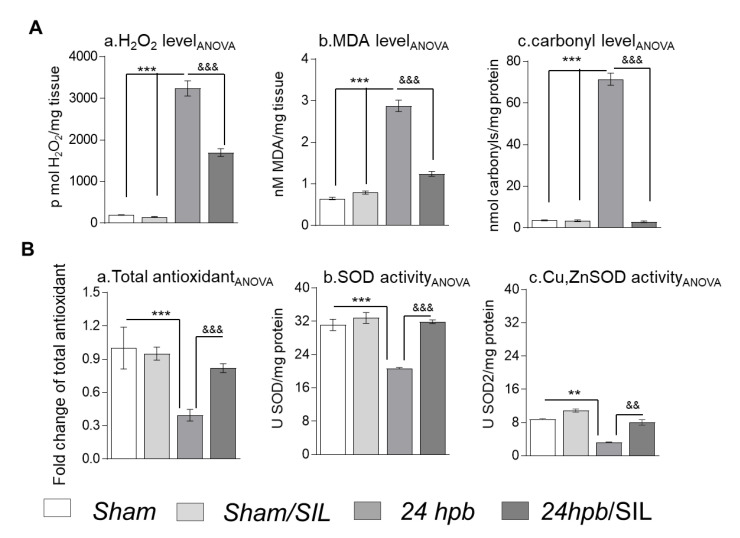
The effect of PDE5 inhibition on oxidant/antioxidant imbalance. Sprague–Dawley rats were randomized to a 60% TBSA scald burn or sham procedure with standard resuscitation with/without sildenafil (SIL). Heart tissues and blood were harvested at 24 h post burn (24 hpb ± SIL). (**A**) Shown are H_2_O_2_ levels (a), MDA levels (b) and protein carbonylation (c) in myocardium after burn. (**B**) Shown are cardiac antioxidants, including total antioxidant (a), total SOD activity (b) and Cu,ZnSOD activity (c) after burn. Results were normalized to rat GAPDH and β-actin mRNAs, and represent fold change after burn (±SIL), as compared to that noted in matched normal controls. In all figures, data are plotted as mean value ± SD. Significance is shown as * (24 hpb vs. control) or ^&^ (24 hpb vs. 24 hpb/SIL), and presented as **^, &&^ *p* < 0.01, ***^, &&&^ *p* < 0.001 (n = ≥ 6 per group).

Our recent publication [13] revealed that burn injury resulted in cardiac mitochondrial damage and transcription factor regulation of antioxidant gene expression, leading to oxidative stress. In this study, we measured oxidative stress markers to determine the role of PDE5 inhibition on ROS generation and the oxidant/antioxidant balance. We found a 16-fold increase in H_2_O_2_ (Figure 6(Aa)), a 3.4-fold increase in malondialdehyde (MDA; Figure 6(Ab)), and a 19-fold increase in the protein carbonyl level (Figure 6(Ac)) after a burn. Sildenafil treatment partially recovered H_2_O_2_ (50.59%) and MDA levels (73.33%), and completely normalized protein carbonyl levels (Figure 6A). Total antioxidants (Figure 6(Ba)), total SOD activity (Figure 6(Bb)), and Cu,ZnSOD (Figure 6(Bc)) showed declines of 60%, 87%, and 22% after a burn, respectively. Sildenafil administration normalized antioxidants, including total antioxidants (Figure 6(Ba)), total SOD activity (Figure 6(Bb)) and Cu,ZnSOD activity (Figure 6(Bc)). These observations suggest that sildenafil not only inhibited PDE5A, but was also involved in the positive regulation of either total or mitochondria specific antioxidants.

## 4. Discussion

In this study, we hypothesized that the NO-PED5-cGMP-PKG pathway plays a very important role in cardiac dysfunction after a burn injury, and that sildenafil, a PDE5A inhibitor, would ameliorate burn-induced cardiac dysfunction. Previous research demonstrated that the NO-PDE5A-cGMP-PKG pathway can preserve heart function and cardiomyocyte mitochondrial function through PKG1α kinase activation [21], and that PDE5A is upregulated in the hypertrophied heart [22,23]. Our data demonstrate that sildenafil protects against heart fibrogenesis and failure after a burn, prevents PKG1α kinase activity deficiency, increases ROS scavenging capacity, preserves oxidative protein adducts and abrogates cardiomyocyte inflammatory infiltrate after a burn. To the best of our knowledge, this is the seminal study demonstrating that the PDE5A-cGMP-PKG pathway plays a central role in burn-induced heart dysfunction. This study is also the first to demonstrate that sildenafil preserves antioxidant scavenging capacity while arresting the oxidative and inflammatory infiltration that causes cardiomyocyte death and cardiac remodeling after a burn. Additionally, we propose that currently available PDE5A targeting drugs, such as sildenafil, might be useful in the treatment of patients with burn-induced cardiac dysfunction.

Previously published articles have shown that sildenafil is effective in numerous heart-related diseases, such as diabetes [24], Chagas disease [25], heart failure [26], hypertension [27] and burns [14]. Our previous work has demonstrated that a burn induces cardiac mitochondrial damage, as evidenced by morphological changes on electron microscopy, cardiac mitochondrial replication deficiency, and decreased mitochondrial complex activity and oxygen consumption [14]. Interestingly, treatment with sildenafil preserved the mitochondrial structure, respiratory chain efficiency and energy status after a burn [14]. This previous work indicates that PDE5A inhibition could be beneficial in treating burn-induced heart dysfunction. However, the complete mechanism remained unclear, as did the question of clinical improvement. This is the first study to demonstrate that the use of a PDE5A inhibitor leads to functional improvement in burn-induced cardiac dysfunction, and that cardiac fibrosis, inflammation and oxidative stress can be mitigated as well.

Heart damage is a well-documented complication that enhances mortality and morbidity after a severe burn injury [28]. However, little is known about the role of the PED5A-cGMP-PKG pathway in burn-induced cardiac dysfunction. PDE5A activation depresses cGMP and PKG levels, whereas PDE5A inhibition would do the reverse [29]. Furthermore, cGMP converts inactive PKG to activated PKG [9], resulting in phosphorylation of the cell membrane and a decrease in cell membrane-bound protein Ras homolog member A (RhoA) activation. This adjusts Rho kinase (ROCK), RGS2 and myosin phosphatase targeting subunit (MYPT) [30]. PDE5A inhibition raises cGMP levels, leading to downstream effects on the heart and vasculature [31]. PDE5A inhibitors may also inhibit RhoA-Rho kinase [14]. In this study, sildenafil was seen to not only inhibit PDE5A, but also to affect PKG-regulated genes. Given this finding, future studies will examine whether sustained PKG activity would protect against burn-induced heart dysfunction.

Severe pediatric burn injury has a long-term effect on heart function into late adolescence and beyond, and is associated with myocardial fibrosis [32]. To test whether burn injury-induced heart function correlates with cardiac fibrosis, we measured heart fibrogenesis by staining heart tissues with trichrome. Our findings indicate that burn-induced heart dysfunction is associated with heart fibrogenesis, and that this process begins immediately after the initial burn injury. Interestingly, PDE5A inhibitor treatment ameliorated burn-induced myocardial fibrogenesis. Histologic and molecular studies demonstrated that burn injury results in cardiac fibrosis, widespread inflammatory infiltration and oxidative adducts in the heart tissue, all of which are common hallmarks of heart dysfunction. While the cause of cardiac dysfunction after a burn remains elusive, our results demonstrate that treatment with sildenafil has cardioprotective effects via preservation of systolic function after burn. We observed decreased heart wall thickness and fibrosis with sildenafil treatment after a burn, which may indicate a lasting protection against burn-induced cardiac dysfunction.

The canonical NF-κB pathway is activated by proinflammatory cytokines, resulting in the activation of RelA-containing complexes, which has an important role in the pathogenesis of chronic inflammatory diseases [33]. In burn-induced cardiac dysfunction, there are no previous studies that demonstrate the presence of RelA-containing complexes. Our data are the first to show that RelA expression in heart tissue is required for the recruitment of NF-κB after a burn (Figure 4(Da)), demonstrating that the canonical pathway of NF-κB plays a very important role in burn-induced inflammation. IL-18 primarily facilitates Th1-type immunoreactions, and helps to generate an inflammatory response [34]. Because IL-18 regulates the synthesis of TNF-α, IL-1β, IL-8 and MIP-1α, removal of IL-18 may have a beneficial effect in lethal endotoxemia in naive mice [35,36,37,38]. Previous studies also suggest that either mRNA or protein levels of IL-18 are enhanced in heart disease and heart infarction, and that IL-18 or IL-1β play a significant role in myocardial injury.

In ischemic cardiac disease, TGF-β neutralizes macrophages through Smad3-dependent pathways [39]. TGF-β may act as one of the key molecules in hypertrophy and heart failure, but its role in burn injury has yet to be elucidated [40]. Our study shows that burn injury increased TGF-β gene expression, and that the TGF-β system may be a promising therapeutic target for burn-induced cardiac dysfunction in the future.

PKG has pleiotropic physiological functions in the cardiovascular system [41]. Specifically, in terms of cardiomyocyte contractility, the substrates of PKG include myosin phosphatase subunit 1 (MYPT1), G-protein signaling 2 (RGS2), phospholamban (PLB) and inositol trisphosphate receptor-associated cGMP kinase substrate protein (IRAG), which demonstrates a functional regulation by PKG. To test the effects of a burn on the PKG pathway, we applied qPCR to measure related mRNA levels, and found significant decreases in PKG, IRAG, PLB, RGS2 and MYTP, as well as an increase in RhoA (Figure 5). This indicates that burn-induced heart dysfunction is mediated via the PKG pathway, and that PDE5 inhibition is beneficial to preserve cardiac function after a burn injury.

ROS are correlated with enhanced PDE5A mRNA levels in cardiomyocytes during heart damage [42], but the mechanism remains unknown. Similarly, little is known about the influence of PDE5A inhibitors on oxidative damage and its association with the activation of cGMP-PKG. However, enhancement of cGMP may reduce NADPH oxidase expression or activity and, therefore, ROS production [43]. Our study shows that PDE5A is a key factor of the compromised antioxidant response, and that sildenafil treatment accelerates ROS scavenging capacity via activating SOD and enhancing total antioxidants. These findings uncover the fundamental cause for the imbalance between oxidants and antioxidants after a burn. The potentially therapeutic benefits of PDE5A inhibitors seem to be the host’s capacity to avoid risks related to extreme levels of O_2_^−^ that result in the overproduction of free radicals. Future studies will aim to illuminate whether PDE5A inhibits antioxidant capacity by targeting the PGC-1-α/NFE2L2 (Nuclear Factor, Erythroid 2 Like 2) pathway of antioxidant response in the heart.

There is also significant overlap between the adrenergic and PDE5 pathways, which will require further study. Borlaug et al. demonstrated that sildenafil administration blunts systolic responses to β-adrenergic signaling, and that sildenafil inhibits β-adrenergic–stimulated cardiac contractility in humans [44]. Additionally, other work has demonstrated that PDE5 inhibition restores catecholamine responsiveness and partially reverses contractile dysfunction [45]. A burn stimulates much higher levels adrenergic proteins [46] and catecholamines [47] in the circulation. Given the interaction between these two systems, further study is needed to further define the interactions between these two pathways in burn-induced cardiac dysfunction.

## 5. Conclusions

Burn-induced cardiac dysfunction occurs via the PDE5A-cGMP-PKG pathway. The PDE5A inhibitor sildenafil has a potent cardio-protective effect, and acts by inhibiting cardiomyocyte inflammation, fibrogenesis and oxidative stress after a burn.

## Figures and Tables

**Table 1:** Oligonucleotides used for study of the MIT copy number.

Gene	5′-Forward-3′	5′-Reverse-3′	Ampliconsize (bp)	Accession #
α-tubulin	CAATTCCATCCTCACCACCCA	TCAACCTGTTTAAGTTAGTGTAGGT	137	AH002269.2
β-actin	ACTGGCATTGTGATGGACTC	GCTCGGTCAGGATCTTCATG	142	V01217.1
ACTA	AGGGAGTGATGGTTGGAATG	GGTGATGATGCCGTGTTCTA	110	BC158550.1
ANP	CGAGAGTCAGGAAACGGAAAG	CTCAGACACACACACACATACA	99	M60731.1
BNP	CACCTCTCAAGTGATCCTGTTT	GCAAGTTTGTGCTGGAAGATAAG	99	M25297.1
COLIA2	CCAGAGTGGAAGAGCGATTAC	ATGCAGGTTTCACCAGTAGAG	101	NM_053356.1
COLIIIA1	CAGGCCAATGGCAATGTAAAG	GCCATCCTCTAGAACTGTGTAAG	108	BC087039.1
GAPDH	ACTCCCATTCTTCCACCTTTG	CCCTGTTGCTGTAGCCATATT	105	NM_017008.4
IL-18	GAATCCCAGACCAGACTGATAAT	GGTAGACATCCTTCCATCCTTC	96	NM_019165.1
IRAG	GTACAACTGTCCTTGGCCTTTA	GTTCCTTCTCGGTGTTCTCTTC	106	NM_001105211.1
MYTP	CCTTTCCAGCACAAGCACTA	GTACTATCCTCAGCCCACAAAC	99	AF110176.1
PDE5	GCCGCCACTATTATCTCCTTC	CTACTTCCTCCCACTCCATTTG	112	NM_133584.1
PKG	GGGAAGGTCGAAGTCACAAA	CTGTCCGGGTACAGTTGTAAAG	100	EU251189.1
PLB	TATCAGGAGAGCCTCGACTATT	CAGATCAGCAGCAGACATATCA	105	NM_022707.2
RELA	GCTCAAGATCTGCCGAGTAAA	GTCCCGTGAAATACACCTCAA	113	AY307375.1
RGS2	GGAAGACCCGTTTGAGCTATT	TCCTCAGGAGAAGGCTTGATA	106	NM_053453.2
RhoA	GACCAGTTCCCAGAGGTTTATG	GTCCCATAAAGCCAACTCTACC	96	D84477.1
αSMA	CACCGCTGAACGTGAAATTG	CTTCTCCAGAGAGGAGGAAGAT	109	NM_019183.1
TGF-β	CTGAACCAAGGAGACGGAATAC	GTTTGGGACTGATCCCATTGA	106	AY550025.1
